# Geochemistry of Gold Ores Mined During Celtic Times from the North-Western French Massif Central

**DOI:** 10.1038/s41598-019-54222-x

**Published:** 2019-11-28

**Authors:** Sandrine Baron, Călin G. Tămaș, Marion Rivoal, Béatrice Cauuet, Philippe Télouk, Francis Albarède

**Affiliations:** 1 Centre National de la Recherche Scientifique, Laboratoire ≪ Travaux et Recherches Archéologiques sur les Cultures, les Espaces et les Sociétés ≫, UMR 5608, Université de Toulouse, Maison de la Recherche, 5 allées Antonio-Machado, 31 058 Toulouse, Cedex 09 France; 2 Department of Geology, Faculty of Biology and Geology, University Babeş-Bolyai, Str. M. Kogălniceanu nr. 1, 400084 Cluj-Napoca, Romania; 3 Ecole Normale Supérieure de Lyon, Université de Lyon, Centre National de la Recherche Scientifique, UMR 5276, 69364 Lyon, Cedex 07 France; 4 Centre National de la Recherche Scientifique, Laboratoire de Planétologie et Géodynamique, UMR 6112, Université de Nantes, 2 rue de la Houssinière, BP 92208, 44322 Nantes, Cedex 03 France

**Keywords:** Solid Earth sciences, Geochemistry

## Abstract

The Celtic culture of Western Europe left magnificent gold objects, such as jewellery and weapons from nobility graves and hoarded coins, as well as field evidence of pre-Roman gold mining and metallurgical workshops that attest to the mining of local ores. This is the case of Central France where many precious metallic ores have been mined throughout the ages from the Prehistoric times onwards. One of the lingering problems in assessing the provenance of gold artefacts and coins is the lack of relevant data on the isotope geochemistry and mineralogy of ore sources. Forty gold ores samples were collected and studied from Limousin (French Massif Central), a very significant gold mining district from the Celtic times. Their Pb isotope compositions clearly show a local dichotomy *i.e*. two distinct groups of ores, one of Late Proterozoic to Early Paleozoic Pb model age and another associated to Variscan ages and consistent with field relationships, mineralogy and elemental analyses. The use of Cu and Ag isotopes, and their coupling with Pb isotopes, will refine the tracing of future metal provenance studies, but also highlight some metallurgical practices like deliberate metal additions to gold artefact or debasement of gold coins. The newly acquired Pb, Ag, and Cu isotopic data on gold ores improves our understanding of ore deposits geology and provide clarifications on the provenance of Celtic gold from this area and its economic importance.

## Introduction

Gold and silver coins and gold artefacts from early Bronze Age to Antiquity have been discovered in burial sites and hoards throughout Europe^1–3^. Archaeometallurgists evaluate the role of these metals in human societies from two complementary perspectives: i) the chain of manufacturing technologies from mines to artefacts^4–6^, and ii) the provenance of ores which provided the metal used to manufacture a particular artefact^7–11^. Manufacturing is key to the understanding of alloying techniques, monetary practice, in particular debasement^12,13^, while assessing the provenance of ores can reveal significant information on ancient sources of wealth and on routes along which metals were traded.

Establishing the provenance of gold is a particular challenge in archaeometallurgy for several reasons. Historical texts, where they exist, provide only a fragmented view on ancient mining and metallurgy, and the locations of many ancient mining sites are not recorded in preserved texts or went unrecorded^14^. Several gold ore deposit provinces are known in Europe, *e.g*., the Iberian belt, the Massif Central of France, the Carpathian belt with notably the Apuseni Mountains in Romania. Most existing provinces focus on ore mineralogy and genetic interpretations and very few of them explore the archaeological aspects. Thus, the community of researchers working on the provenance of gold use these available mining geological data because of the lack of mining and/or metallurgical referentials previously studied by archaeology. Many historically important mining areas, such as the Iberian Pyrite Belt, have experienced multiple periods of activity extending even to the present day, which makes assessing archaeological contexts difficult^15^. The original size of each mined ore deposit and its mining history is usually not known with precision^2^. When evidence for multiple periods of mining activity in a single mining site, notably during Roman and Medieval times, is occasionally revealed by archaeological work, the record is usually patchy and ambiguous. However, detailed geochemical analyses of ores from mines previously excavated and dated by mining archaeology can lay the foundation for providing better assessments of the provenance of artifacts at regional scale. Indeed, only part of an ore deposit was exploited by the ancients as demonstrated by archaeological excavations^16–20^ and exploitation resumed frequently. There are two main reasons for this *i.e*. i) the legal/administrative status of the mines for a given historical and cultural context and ii) the deliberate technical choice of the ancients. A mine could be public or private and involve different terms and conditions of mining depending on its status. Some specific ore(s), within the same mine/mining district, was/were extracted according to several factors such as mining management (concession mode or not), economical and geopolitical reasons, mining knowledge and technical skills at a given period, and constrained by the shape and the mineralogy of the ore body(ies). All these factors related to the mining together with the ore processing and metallurgical approaches had an impact on the signature of the metal production at the mine/mining district scale. In addition, a mining district has a complex geological history and the isotopic signatures of the ores from different ore bodies/ore deposits are rarely homogeneous. Consequently, the coupling of anthropogenic and geologic controls lead to the need for more accurate characterization that becomes relevant for archaeological and historical applications^21^. The data available in the geological literature is not precise enough for these purposes because it is not representative for the ores actually exploited by the ancients. Nevertheless, this data is very useful in order to place a particular study area in a given geodynamic context and to reject some geographical ore sources, but it does not provide an accurate geochemical signature for regional archaeological and historical issues. The present work combines mining archæology (for which the results were acquired through previous research project)^4,16,22^ with original data on mineralogy, and geochemistry with high-precision measurements of Pb, Cu, and Ag isotopes abundances to characterize this major Au-Ag primary ore district mined from 6^th^ to 1^st^ c. BC^4,16,22^ in Limousin (western French Massif Central).

The French Massif Central was the most productive French Variscan metalliferous province and hosts various ore deposit types^23,24^. Some of these ore deposits were mined during the Antiquity, mostly for gold, silver, lead, and tin. Important mining operations developed during the 19th and 20th centuries mostly for uranium, tungsten, antimony and gold. Gold mining in Limousin has so far yielded about 45 tons of gold metal^25^. Other industrials minerals, such as barite and fluorite, were also extracted.

**Figure 1:**
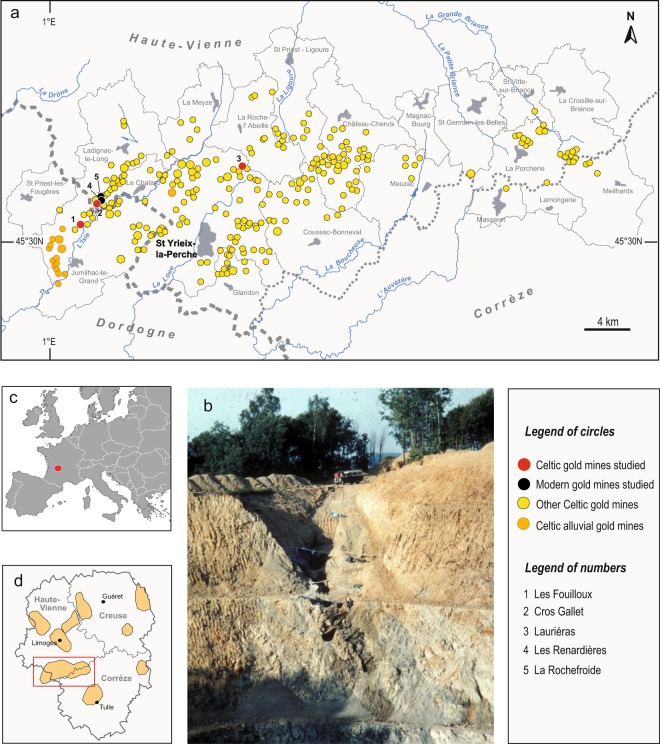
(**a**) Detail of the gold mines in the Saint Yrieix-la-Perche district; (**b**) The Celtic gold mine of Les Fouilloux (one among several others mines) with its underground wooden equipment cut by the modern opencast mine; (**c**) Limousin area in Europe; (**d**) Celtic gold mines inventory in Saint Yrieix-la-Perche district, Limousin (French Massif Central). DAO maps produced under Adobe Illustrator CS6 and picture are from B. Cauuet.

More than 250 Prehistoric gold mining sites (Fig. 1a), including over 1100 opencast individual mines operated by the Celtic tribe known as Lemovices were identified in Limousin^16,26^ (Fig. 1b–d). Mining archaeological investigations^4,16,22,27^ enabled ancient mining to be traced back to the Iron Age, with evidence of exploitation as early as the 6th to the 3rd c. BC and showed that the mining activity climaxed during the 2nd to 1st c. BC. The mining archaeological diggings^22^ (Fig. 1b) confirmed that the ancient mining activity came to an end at the time of the Roman conquest (mid- to late 1st c. BC). The cumulated metal production of Limousin from the 6th to late 1st BC was estimated to be over 74 tons of gold. This estimate takes into account an average ore grade of 20 g/t Au and a yield of 90%, based on archaeological experimentations^16,26^. By comparing the gold tonnage in relation to the area, the relative Celtic production exceeds the estimated Roman one from the north-western part of the Iberian Peninsula^15^.

This paper’s objective is to provide a geochemical and mineralogical background, including Pb, Cu and Ag isotopic compositions on ore samples collected at surface and in underground mining workings known to have supported gold metal production during Celtic times (Lemovices tribe). However, ores mined during the modern times were also included in the study. Our expectation is that the new body of data will provide a firm reference for archaeometallurgical studies of Celtic gold, while adding new elements for the understanding of ore deposits in Western Europe.

## Geological Setting

Several Variscan ore-forming events relevant to gold deposits have been identified in France^24,28,29^. The first event, the Gold-400 Ma, is well documented in Brittany, and is represented by base-metal sulfides and electrum. The second event, named Gold-360 Ma, is represented by Zn-Cu-Pb-Ba massive sulfides deposits, locally associated with precious metals in Visean basins. Two further metallogenetic events took place at the end of the Variscan orogeny. The early event is best developed in the Cevennes, south-eastern Massif Central. It is dated around 350–325 Ma, but does not contain gold. The latest event was recognized in the Limousin, north-western Massif Central. It has a clear Au-Sb overall geochemical character and is known as the Gold-300 Ma event. Most of the French gold deposits belong to this metallogenic event^24^.

## Geology of the Studied Area and Samples Location

**Figure 2:**
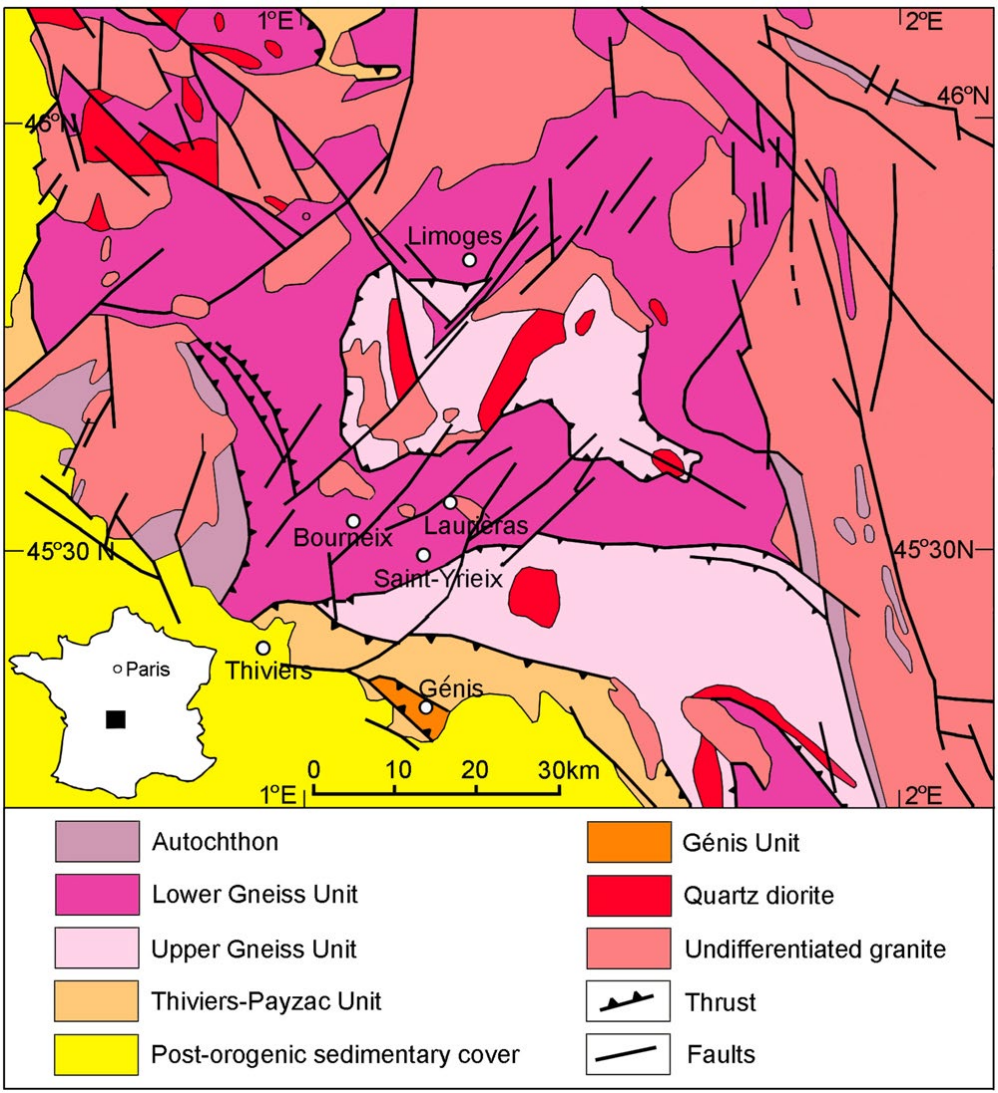
Geological map of the studied area. The map was simplified from the original geological map of France (J. Chantraine, A. Autran, C. Cavelier, Geological map of France at a 1/1 000 000 scale, BRGM, Orléans, France, 1996). DAO map produced under Adobe Photoshop CS5.1 by C. G. Tămaș. The original geological map can be viewed on the InfoTerre website, which is a free access service offered by the French Geological Survey (http://infoterre.brgm.fr/viewer/LoadContext.do?uuid=cc5b530f-ddf4-464b-9045-2667167bbfbf). The terms of use are reported here (https://www.brgm.eu/scientific-output/digital-data-services/infoterre-portal-access-to-brgm-geoscientific-data).

There are three main gold ore districts in the French Massif Central, i.e. St Yrieix (Saint-Yrieix-La-Perche), Salsigne and Marche-Combrailles^25^. Most gold ore deposits mined by the Celts in Limousin occur in the St Yrieix district, 40 Km south of Limoges (Fig. 1c,d). This district (Fig. 1) was one of the latest active gold mining areas in Western Europe. The western French Massif Central is composed of several metamorphic units of Late Proterozoic to Early Paleozoic age stacked together by the Meso-Variscan collision (Devonian to Early Carboniferous)^30^. From bottom to top, the main lithological units are the autochton basement, the Lower Gneiss Unit, and the Upper Gneiss Unit^31^ (Fig. 2). Both the autochthon basement and the Lower Gneiss units comprise amphibolite-grade paragenesis, micaschists and orthogneisses. The protolith of the Lower Gneiss unit is composed of Cambrian to Ordovician granitoids. The lithology of the Upper Gneiss Unit is more complex and its protolith formed during the Cambrian and the Silurian in an oceanic realm and it consisted of sediments and volcanics^30^. During the Neo-Variscan stage (~330–290 Ma), the French Massif Central experienced an interplay of compressional and extensional tectonics and large-scale granite emplacement^30^. Overall, the ore deposits formed at the apex of subjacent granitic intrusions and dominantly belong to the As-Sb association^24^. They are structurally controlled by shear zones. The gold deposits were formed by an intense hydrothermal activity^32^. They generally consist of quartz lenses and veins about 10 s to 100 s meters long and up to ten meter thick^24,33^. The ore minerals are represented by native gold associated with sulfides (pyrite, chalcopyrite, stibnite, sphalerite, arsenopyrite) and various sulfosalts (tetrahedrite, pyrargyrite, boulangerite, bournonite, semseyite) within abundant massive quartz gangue. Two main Variscan ore deposition events were separated in France^24,33^. The early event is characterized by the presence of arsenopyrite, pyrrhotite, pyrite, gold and Pb-sulfosalts, whereas the late event is characterized by the presence of sulfides and sulfosalts and abundant Ag and Bi.

The ancient and modern mining sites investigated in the present work are located in the western part of the St Yrieix district (Figs. 1 and 2). The sampling of the archaeological ores in the field was done according to a interdisciplinary archaeological and geological approach^21^. Previous field work revealed traces of ancient mining activity in several ore deposits in Limousin^16^. Some of these ancient mines became partly accessible during and after the modern open-pit operations carried out in the 1980s by the French mining company COGEMA. The presence of a Celtic mining activity was confirmed by Cauuet and co-workers^4,16,22,27^ and our geological field work allowed for the sampling of various types of ores exploited by the ancient and modern miners.

The St Yrieix ore district consists of two distinct metallogenetic groups collectively referred to as Le Bourneix and Lauriéras^ 34^.

### Le Bourneix Mines

In this group, we investigated the mines named, from south to north: Les Fouilloux, Cros Gallet, Les Renardières, and La Rochefroide (Fig. 1a). The Lauriéras site is located about 10 km east of the Le Bourneix group^34^. Ore samples were collected from two Celtic mining sites (Les Fouilloux and Cros Gallet) (Fig. 1a). Samples from several modern mines, *i.e*., part of Cros Gallet, Les Renardières and La Rochefroide were also collected as reference to validate our approach.

The Les Fouilloux samples come from a pillar of a Gaul mine. The Les Renardières and La Rochefroide samples were collected from the modern open pit.

### Lauriéras Mines

The samples from Lauriéras include ore fragments collected from the modern open pit. They were picked up during mining archaeological diggings of the old mining sites, or from the ancient open pit still preserved on the northern edge of the modern pit.

## Results

### Gold ores mineralogy

Several methods were used to characterize the geochemistry and mineralogy of the gold ores, which included analyses of Pb, Cu and Ag isotopes, reflected light microscopy, scanning electron microscopy (SEM) and electron probe microanalysis (EPMA). The mineralogical results are summarized in Table 1 and the Pb, Cu and Ag isotopic compositions of ores (bulk analyses) are reported in Table 2. The label ‘A’ refers to the gold ore samples exploited by the ancient miners and the label ‘M’ to those of the modern mining sites (open pits/underground).

**Table 1:** Summary of the mineralogy of primary Au-Ag ores from Limousin (French Massif Central).

Ore deposit/	Ore grades (ppm)				Ore Mineralogy (*)
mining site	Sample Id	Au	Ag	Pb	
Les Fouilloux (A)	4300	30.6	6	233	stibnite, pyrite, arsenopyrite, Ag-poor tetrahedrite (4.5–4.8 wt % Ag), chalcopyrite, galena, sphalerite, berthierite, jamesonite, gold with Au/Ag ratio: 86/14–80/20
	4301	56.4	11.5	156	
	4302	80.6	9.3	148	
	4303	39.9	28.3	2890	
	4306	94.0	9.4	401	
	4453	82,03	17.4	308	
Cros Gallet (A)	4438	306.89	16.3	104	gold, arsenopyrite, lindstromite, native bismuth; two gold populations (Au/Ag ratio): 89/11–87/13 and 81/19
	4141	na (**)	na	na	
	4142	na	na	na	
Cros Gallet (M, underground)	4439	1105.85	45	624	gold (Ag = 28 wt%), arsenopyrite, pyrite, galena, chalcopyrite, sphalerite, bournonite and boulangerite
Cros Gallet (M, open pit)	4315	0.31	<0.5	11	pyrite, arsenopyrite
	4316-a	0.27	<0.5	9	
	4320-a	0.15	<0.5	6	
	4321-a	0.16	<0.5	12	
	4322	0.33	<0.5	10	
	4323	0.23	<0.5	12	
	4324	0.20	<0.5	11	
	4327	0.34	<0.5	12	
	4330	0.66	<0.5	18	
Lauriéras (A)	4532	0.32	5.2	171.74	pyrite, arsenopyrite, boulangerite/jamesonite, chalcopyrite traces
	4534	0.87	2.4	491.87	
	4536	1.78	2.1	724.68	
	4538	0.22	0.1	<L.D.	
	4540	0.03	0.2	5.88	
Lauriéras (A/M)	4363	0.03	0.7	48	pyrite, arsenopyrite
	4366-a	0.17	7.8	337	
	4358-a	9.14	80.5	>10000	
	4541	12.64	5.5	26.88	
	4537	0.22	0.46	19.39	
Lauriéras (M)	4143 A	na	na	na	type 1 gold (Au/Ag = 87/13) with Ag-poor tetrahedrite, (4 wt% Ag), bournonite, boulangerite, jamesonite, galena, sphalerite, chalcopyrite and arsenopyrite
	4143 C	na	na	na	
	4143D	na	na	na	
	4150	na	na	na	
Lauriéras (M)	4144B	na	na	na	type 2 gold (Au/Ag = 51/49) with Ag-rich tetrahedrite (17 wt% Ag), sphalerite, chalcopyrite, galena, pyrite, and arsenopyrite
	4144 C	na	na	na	
Lauriéras (M)	4149B	na	na	na	type 3 gold (Au/Ag = 75/25) with arsenopyrite
	4149 C	na	na	na	
Les Renardières (M)	4337	0.28	1.2	15	gold (Au/Ag = 65/35) with Ag-rich tetrahedrite (10–17 wt % Ag), pyrite, sphalerite, chalcopyrite, arsenopyrite, galena, boulangerite, jamesonite, freibergite, acanthite
	4342	0.22	1.3	97	
La Rochefroide (M)	4349	0.47	1.9	91	pyrite
	4350	0.94	5.5	100	
	4351	0.93	2.9	201	

The label (A) means Ancient ores, and the label (M) means Modern ores. (*) Stibnite: Sb_2_S_3_; Pyrite: FeS_2_; Arsenopyrite: FeAsS; Chalcopyrite: CuFeS_2_; Galena: PbS; Sphalerite: ZnS; Berthierite: FeSb_2_S_4_; Jamesonite: Pb_4_FeSb_6_S_14_; Lindstromite: Pb_3_Cu_3_Bi_7_S_15_; Boulangerite: Pb_5_Sb_4_S_11_; Bournonite: PbCuSbS_3_; Freibergite: (Ag,Cu,Fe)_12_(Sb,As)_4_S_13_; Acanthite: Ag_2_S; Tetraedrite: (Cu,Fe)_12_Sb_4_S_13_.

(**) Not analysed.

**Table 2:** Pb, Cu and Ag isotopic data on primary gold ores from Limousin Celtic mining district (accuracy and precision are reported in the main text and diagrams).

Samples Id	^206^Pb/^204^Pb	^207^Pb/^204^Pb	^208^Pb/^204^Pb	δ ^65^Cu ^(#)^	ε ^109^Ag	1sd
**Les Fouilloux A**						
4300	18.217	15.625	38.364	−0.397	−0.283	*0.100*
4301	18.232	15.609	38.327	−0.389	0.013	*0.058*
*duplicate*	18.229	15.606	38.316	na ☨	na	—
4302	18.214	15.591	38.273	−0.386	−0.291	*0.072*
*duplicate*	18.211	15.589	38.265	na	na	—
4303	18.053	15.580	38.124	−0.708	0.029	*0.050*
4306	17.993	15.580	38.099	−0.636	n.a	—
4453	18.036	15.594	38.165	0.409	−0.251	*0.044*
**Cros Gallet A**						
4438	18.058	15.583	38.166	−0.620	−0.150	*0.008*
**Cros Gallet M**						
4439	18.076	15.600	38.208	0.062	−0.246	*0.029*
4315	18.906	15.625	38.724	−0.549	na	—
*duplicate*	18.901	15.621	38.713	na	na	—
4316	19.122	15.628	38.698	−0.416	na	—
*duplicate*	19.121	15.630	38.697	na	na	—
4320	18.971	15.616	38.867	−0.135	na	—
*duplicate*	18.975	15.620	38.876	na	na	—
4321	19.229	15.627	38.540	−0.205	na	—
*duplicate*	19.227	15.627	38.536	na	na	—
4322 (host rock, gneiss)	18.637	15.597	38.374	−0.219	na	—
4323 (host rock, gneiss)	18.752	15.608	38.478	−0.063	na	—
4324	18.879	15.613	38.553	−0.212	na	—
4327	18.908	15.614	38.561	−0.358	na	—
4330 (host rock, gneiss)	18.593	15.600	38.342	−0.574	na	—
**Lauriéras A**						
4532	18.197	15.602	38.291	0.039	na	—
4534	18.133	15.564	38.412	−0.040	na	—
4536	18.141	15.253	38.586	0.182	na	—
**Lauriéras A/M**						
4363	18.213	15.579	38.220	0.015		
4366	18.186	15.598	38.269	−0.180	−0.240	*0.110*
*duplicate*	n.a	n.a	n.a	−0.154	na	—
4358	18.171	15.644	38.368	−0.067	−0.160	*0.050*
*duplicate*	18.173	15.644	38.366	na	na	—
4541	18.243	15.631	38.370	0.124	na	—
4537 (host rock, gneiss)	19.432	15.679	39.762	−0.136	na	—
4540	18.848	15.647	38.683	0.063	na	—
**Lauriéras M**						
4355*	19.416	15.699	38.664	−0.060	na	—
4538	19.045	15.668	38.789	0.055	na	—
**Les Renardières M**						
4337	18.265	15.616	38.318	−0.030	na	—
4342	18.100	15.608	38.241	−0.110	na	—
**La Rochefroide M**						
4349	18.261	15.625	38.392	−0.475	na	—
4350	18.321	15.622	38.305	−0.755	−0.072	*0.044*
4351	18.193	15.616	38.262	−0.050	−0.129	*0.050*

(#) Typical error of 0.05 per mil (1 sd).

☨: Not analysed.

(*) No ore mineralogy for this sample.

Summarizing the data in Table 1, the ore mineral paragenesis consists of native gold, sulfides (pyrite, chalcopyrite, stibnite, sphalerite, arsenopyrite, etc.), and various sulfosalts (tetrahedrite, boulangerite, bournonite, etc.) hosted by massive quartz gangue. The EPMA analyses on gold grains included in quartz gangue show an elemental gold content >80 wt% and high Au/Ag ratios (Table 1). The Cu grades of the analyzed ores are heterogeneous, ranging from less than 1 ppm to less than 90 ppm (538 ppm Cu for only one sample).

In Le Bourneix mines, Les Fouilloux samples contain abundant stibnite. Gold is Ag-poor and is associated with Ag-poor tetrahedrite, Fe-Sb sulfosalts and quartz (Table 1). Gold from Les Renardières site is richer in silver (about 35 wt%) and is accompanied by Ag-rich tetrahedrite (10–17 wt % Ag), several Pb-Sb sulfosalts, and occasionnally acanthite (Ag_2_S). The La Rochefroide samples revealed a low grade ore with arsenopyrite and pyrite. Three types of ores from the Cros Gallet site were analysed. Several samples were collected during the archaeological diggings and correspond to the ore mined out by the Celts [Cros Gallet (A) - Table 1]. Gold is extremely abundant in these samples and it is clearly visible in quartz gangue. This gold is Ag-poor and it contains inclusions of Bi-minerals. Additionaly, sample 4439 was picked up from a modern underground stope that followed a high-grade ore body not known to the ancient miners (Table 1). This ore contains abundant gold with less than 30 wt% Ag. Several low-grade ore samples from the modern open pit were also analysed [Cros Gallet (M, open pit) - Table 1].

Three types of gold have been identified in the ore samples collected in the modern mines from Lauriéras (Table 1). Type 1 gold is Ag-poor, with an average Au/Ag ratio of 87/13; it is associated with scarce Pb-Sb ± Cu, Fe sulfosalts and Ag-poor tetrahedrite (<4 wt% Ag). Type 2 gold is Ag-rich, with an average Au/Ag ratio of 51/49; it is part of a sulfide-rich mineral assemblage also containing Ag-rich tetrahedrite (>17 wt% Ag). Type 3 gold has an intermediate composition with an average Au/Ag ratio of 75/25 and it is associated with arsenopyrite and pyrite. Apart from pyrite and arsenopyrite, the ores collected from archaeological sites only contain scarce Pb/Fe sulfosalts, even if Au grades range up to 12.64 g/t and Ag grade up to 80.5 g/t (Table 1).

The analysed ore deposits from Limousin are gold-silver ores. Silver is either bound to gold in native gold grains or it is included in Ag-bearing tetrahedrite, while acanthite is rare. The remains of the modern open pits are low-grade Au-Ag mineralizations of no economic importance.

### Pb, Cu and Ag isotopic measurements on gold ores

**Figure 3:**
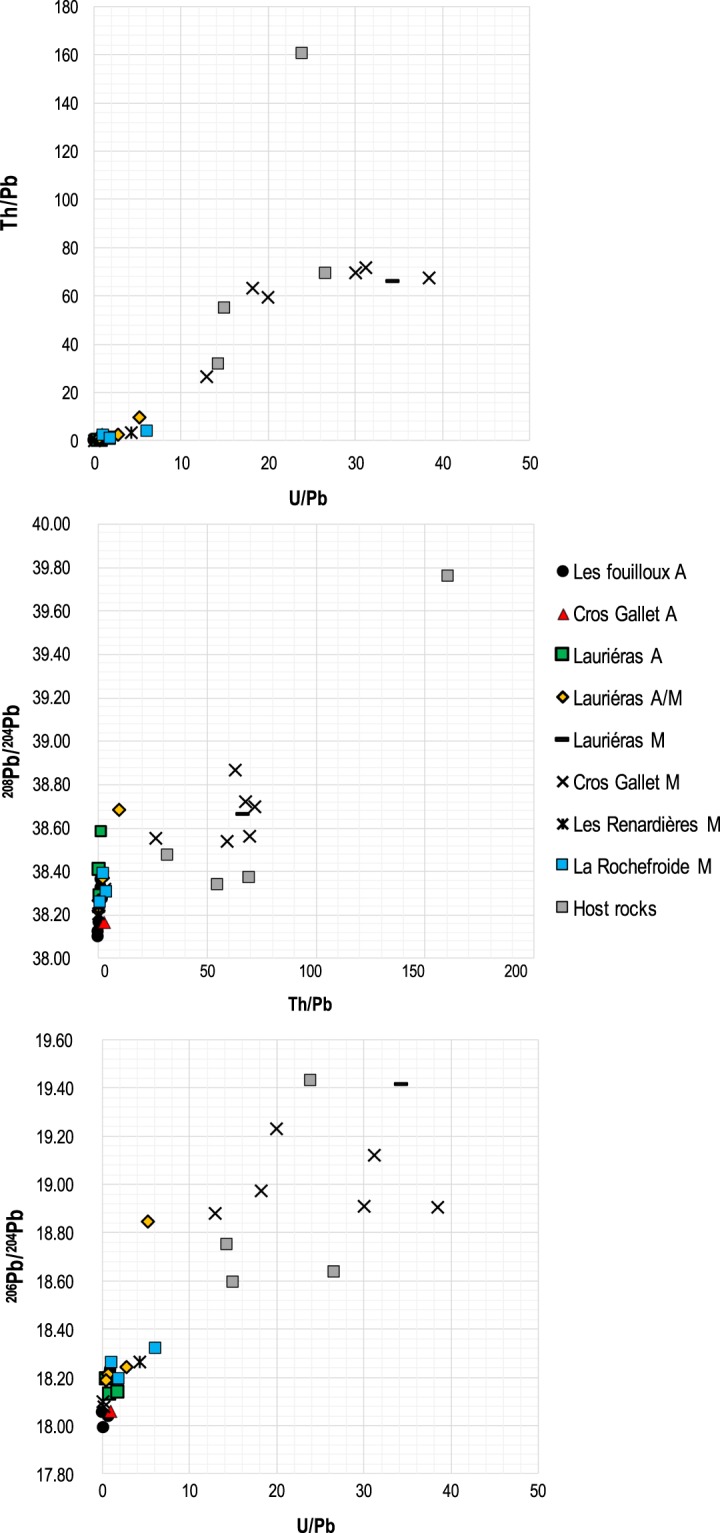
U/Pb versus Th/U, ^208^Pb/^204^Pb versus Th/Pb and ^206^Pb/^204^Pb versus U/Pb diagrams showing the radiogenic ingrowth with geologic times.

With few exceptions, the ^206^Pb/^204^Pb and U/Pb ratios of the gold ores delineate two differents groups (Fig. 3):

#### Group #1

**Figure 4:**
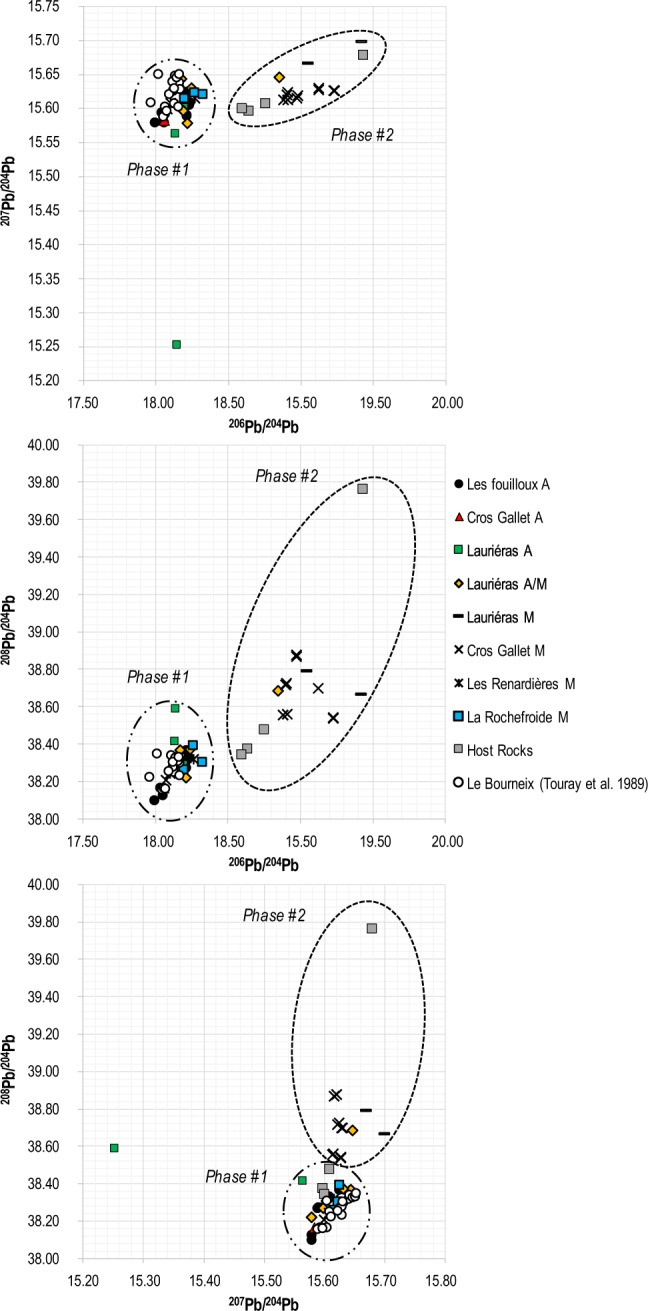
Lead isotopic diagrams of the studied Ancient and Modern gold ores with the Au-bearing lodes and Sn-W occurences studied by Touray *et al*. 1989. Phase #1 corresponds to ores having a U/Pb <1 (Cambro-Ordovician model age); Phase #2 correponds to ores having a U/Pb >1 (Variscan model age).

The ores mined by the Celts in the Les Fouilloux, Cros-Gallet and Lauriéras, and the ores mined recently from Les Renardières and La Rochefroide have low U/Pb (<1) and ^206^Pb/^204^Pb ratios, indicative of Lower Paleozoic model ages (18.0–18.2). The data are quite similar to those pertaining to the nearby Au-bearing lodes and Sn-W occurences of Le Bourneix district, apparently related to Cros Gallet^33^ (Fig. 4). As in Touray *et al*.^33^, the oldest Pb model ages recorded for this group are around 475–500 Ma (Cambro-Ordovician).

#### Group #2

The Cros Gallet ores and other deposits mined during *modern* times have U/Pb >1 and ^206^Pb/^204^Pb ratios in excess of the modern Pb of Stacey and Kramers (1975)^35^; the Lauriéras samples belong to both Groups #1 and #2. The samples from this group are enriched in Al, Na, K, REE, Y, Zr, Hf,Th, Ga, and Nb, and depleted in Pb, Zn, Ag, Bi, and Au as compared to those with Lower Paleozoic Pb model ages. Sample 4536 is anomalous and presents some Cr and REE anomalies.

The ^206^Pb/^204^Pb of host rocks is consistent with modern ‘common’ Pb.

The ε^109^Ag values range from −0.291 to +0.029 and δ^65^Cu from −0.755 to +0.409 (Table 2). The range of ε^109^Ag and δ^65^Cu of ancient and modern ores is comparable (Table 2).

## Discussion

### Model age of gold ores from Limousin (Saint Yrieix-la-Perche district)

A first finding is that ^206^Pb/^204^Pb ratios as low as 18.0–18.2 require a major U/Pb fractionation event taking place before mid-Paleozoic. The Cambro-Ordovician model ages of less radiogenic isotopic signature in Limousin Au-lodes (phase #1) indicate that one source of Pb predates the Variscan orogeny (Fig. 4). These old ages require the existence of Pb sources older than those believed to have formed other Variscan gold ore deposits from the Massif Central^24^. At Le Bourneix, coherent mid-Paleozoic model ages (430–500 Ma) were found^35^, which were interpreted as a mixing between two primary sources. Although mixing cannot be formally discounted, it is clear that the two groups identified in the present work may have acted as end-members with respect to Touray *et al*.’s results^33^. They also provide evidence of two successive generations of ores with distinct events of U/Pb fractionation (Fig. 3).

Whether the dual character of ore deposits reflects the contrasting ages of host rocks, Cambro-Ordovician gneisses and Variscan granite, is not clear. The protolith of the Lower Gneiss Unit was dated to 535 ± 21 Ma by Rb/Sr whole rock^36^, whereas the Upper Gneiss Unit was dated to 525 ± 12 Ma^37^. The Pb model age of gold ores from phase #1 reveals that some gold mineralizations were already present in the waning stages of the Pan-African orogeny. Gold-rich pyrite and arsenopyrite deposits with geological ages consistent with the model ages of Group #1 are known to occur in multiple locations in France, e.g. those hosted by Neoproterozoic rocks, such as the Rouez ore deposit in Brittany, of Brioverian age^24,38^. In the north-western part of the Iberian Peninsula, Pb-bearing minerals from a gold vein system also highlight local unradiogenic signatures^39^. We cannot rule out the possibility that the Group #1 ores actually represent Early Paleozoic ores reworked during the Variscan orogeny, in contrast with Group #2 which indicates a major U/Pb fractionation of Variscan age (Figs. 3, 4). The apparent lack of Variscan deformation in Group #1 ores may lend some support to the reworking hypothesis. The more radiogenic Au ores (phase #2) are comparable to the age of the younger event and consistent with the emplacement of the close-by Variscan two-mica granite of Le Bourneix dated to 301 Ma by K-Ar^31,40–42^. No paragenetic differences between Group #1 and Group #2 stand out. Nevertheless, the spatial relationships observed in the field revealed that the Cambro-Ordovician model age Au-ores at Lauriéras (samples 4366, 4532, 4534, 4536) (phase #1) correspond to the hanging wall of the ore bodies and, occasionally, to locations close to the main fault planes, whereas the ores with Variscan model ages (phase #2) are located in the footwall of the ore bodies or occur as smaller-sized veins hosted in the host rocks, mainly gneisses (samples 4537, 4538, 4540) (Table 1). Moreover, the veins sampled at Les Fouilloux consist of two individual sections separated by a mylonite suggestive of two phases of ore deposition.

### Mineralogy of the gold ores of phase #1 and phase #2

The model age dichotomy among different ores from Limousin is also consistent with ore mineralogy. The ores mined by the Celts at Les Fouilloux, Cros Gallet and possibly partially at Lauriéras belong to the first (older) phase #1. This ore phase is high-grade Au with low Ag contents. The Ag-poor gold of phase #1 is accompanied by Ag-poor tetrahedrite at Les Fouilloux and by Bi-minerals at Cros Gallet. The underground ore (sample 4439) mined during modern times at Cros Gallet also belongs to phase #1 (Fig. 4 and Table 1). In contrast, the low Au grade of the modern ores mined at Cros Gallet and their model ages belong to the younger phase #2. Three types of gold were distinguished at Lauriéras from Au/Ag ratios and mineral paragenesis perspectives (Table 1). This complexity is probably relevant to their large spread of Pb isotope compositions. The first type of gold resembles the Ag-poor ores from Les Fouilloux and is dominated by Ag-poor tetrahedrite. Two other gold types are Ag-rich, with Ag content in gold showing an average of about 50 wt % and 25 wt %, respectively. No isotopic data are available for these ore samples.

EPMA data on ore sample 4337 from Les Renardières (Le Bourneix group) confirm that silver is hosted by gold (30–35 wt % Ag) and by Ag-rich tetrahedrite (up to 17 wt % Ag). The Pb model age of this ore type is Cambro-Ordovician. Likewise, the samples from La Rochefroide (also, Le Bourneix group) reflect mostly the low-grade Au of the host rock that remained after mining, which belongs to phase #1.

A consistent mineralogical interpretation with model ages is that the Au-Ag ores from Limousin (older model age phase #1, Fig. 4) were partially reworked by later hydrothermal fluids during the Variscan orogeny, which led to the formation of new ore bodies. If all the ores may have eventually formed during the Variscan tectonic phase, some of them recrystallized from a pre-existing early-Phanerozoic stock. Phase #1 ores are Au-dominated with Ag-poor gold and Ag-poor tetrahedrite, which, assuming reworking, would indicate that Ag was more depleted than Au in the process. In contrast, the second ores generation (younger Pb model age phase #2; Fig. 4) is enriched in Ag with gold having Au/Ag ratios (in wt%) down to 50/50, and Ag-rich tetrahedrite. A Variscan hydrothermal reworking of the phase #1 ores also accounts for the Au-enrichment of the remaining gold with Au/Ag ratios (in wt %) approaching 90/10. The nature of phase #2 is relatively clear: the low-grade ores from group #2 are enriched in Al, Na, K, REE, Y, Zr, Hf,Th, Ga, and Nb, which point to an albitization process, and depleted in Pb, Zn, Ag, Bi, and Au as compared to group #1. The lower Ag content of the ores with Cambrian-Ordovician Pb model ages would echo ancient observations made on lead ores^43^ that pointed out that silver content of galena (PbS) is higher when model age and geological age are consistent, and lower when model age is older than the geological age of ore deposition.

Hydrothermal mobilization involves Cl- and S-rich fluids. Chloride complexes and bisulfides complexes of gold and silver are among the most effective ligands for the transport of the precious metals in hydrothermal fluids over a large range of thermodynamic conditions^44^. The composition of the deposited native gold/Au-Ag alloys grains depends on complex relationships among the temperature of the hydrothermal fluids, S_2_ and O_2_ activities, Cl^-^ concentration, pH, and the overall Au/Ag content of the hydrothermal fluids^44^. These experimental data indicate that high gold solubility and high Au/Ag solubility ratios reveal low salinity and near-neutral fluids with high dissolved sulfide content, while low gold solubility and very low Au/Ag solubility ratio are characteristic for higher salinity fluids.

### Copper and silver isotopes of the gold ores

Only few δ^65^Cu are available and they are for placer gold localities in Europe^45^ and it is not established at this point that Cu isotopes are a provenance tracer. The use of δ^65^Cu therefore allows us to discriminate between placer gold deposits when Pb isotopes overlap. In addition, Cu istopic comparisons are not truly compelling as the number of samples in each alluvial district is still limited. Heterogeneity of δ^65^Cu in the same province is the rule^8,45–47^, suggesting that the gold samples formed at different depositional stages might be related to different watersheds but also to different primary gold deposits.

The use of silver isotopes studies is still at a very early stage. The first precise Ag isotope analyses in archaeometallurgy were obtained on silver coinage and silver ores, and are opening up new sources of information on provenance^10,48^. Silver isotope compositions on few native gold ores and native silver ones recently measured^49,50^ also raise the issue of the importance of these sources in archaeometallurgy. These native gold and silver mining districts show a much broader range of ε^109^Ag signatures from −8 to + 21.0, which are much more scattered than to gold ores from Limousin mined during Prehistoric times suggesting that low-temperature ores are not involved. Overall, combining Cu, Ag, and Pb isotopes limits the spectrum of possible provenances.

**Figure 5:**
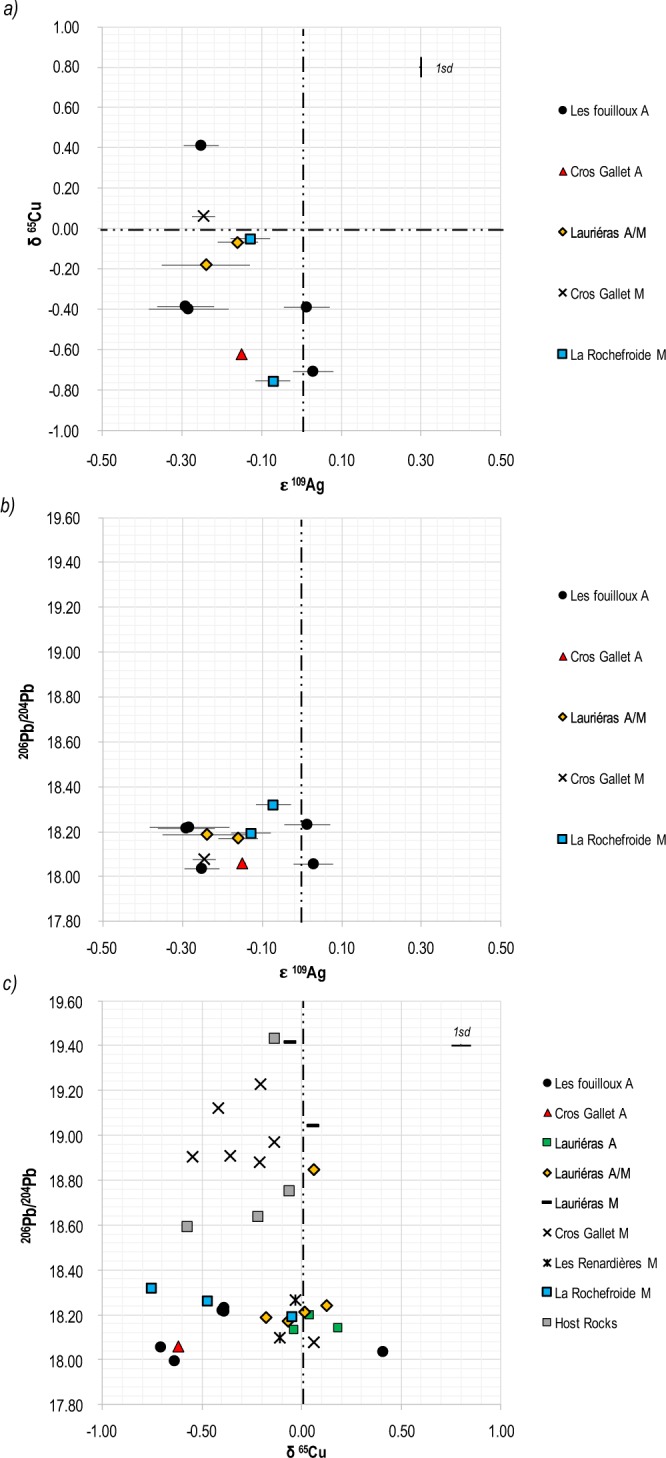
δ^65^Cu versus ε^109^Ag, ^206^Pb/^204^Pb versus ε^109^Ag and ^206^Pb/^204^Pb versus δ^65^Cu diagrams of Ancient, Modern gold ores and host rocks from Saint-Yrieix-La-Perche gold mining district (Limousin, France).

In spite of the limited number of samples measured, Ag and Cu are negatively correlated (Fig. 5a). Overall, the Cu and Ag isotopic compositions of the ancient ores is not specific to Pb age groups (or the ^206^Pb/^204^Pb ratios) (Fig. 5b,c). In contrast to Pb isotopic abundances, which reflect those of the primary gold ore bodies^51^ and the geological history of ores, Cu and Ag isotope compositions largely reflect formation temperatures, and therefore suggest the depth of ores deposition, different redox conditions^49^, and the nature of ligands^52–54^. The range of fractionation should strongly decrease for both Cu and Ag with increasing temperatures. The ores exploited by the ancient miners are hosted in the gossan part of the ore bodies, *i.e*. close to the surface (less than 100 m depth). In this underground area, low-temperature processes, like weathering/leaching favour many oxidation-reduction transformations and thus fractionation processes^47,55–57^. The range of δ^65^Cu of gold ore samples (1.2‰) is consistent with what is known for native copper deposits^58^, but the rather narrow range of their ε^109^Ag (0.04‰) values reflects the fact that most of the silver from the underlying high-temperature deposit has been recycled *in situ*. Therefore, silver seems less mobile than copper in the gossan formation processes. The overall negative correlation between Ag and Cu conditions suggests that Ag and Cu transport and adsorption remain closely connected (Fig. 5a). Copper has three oxidation states (0, 1, and 2) while silver has two (0 and 1). In well-ventilated soils, most sulfur should be oxidized as soluble sulfate and Cu as Cu^2+^. Reductive precipitation of Cu, likely mediated by sulfate reducing bacteria, and buffering of Ag oxidation state by the Cu(0)-Cu^2+^ buffer are a probable mechanism of this negative correlation, but the nature of ligands and the temperature still need to be better understood.

### Archaeological implications

Most studies concerning gold provenance are carried out using elemental analyses focused mainly on gold coins^59–61^. Indeed, major metallic impurities such as Ag, Cu and Sn, which are frequently found in gold coins and artefacts^2^, as well as ultra-trace elements like platinum, iridium and osmium often naturally present in gold ores, have been suggested to carry a valuable source of genetic information^2,7,62–66^. For exemple, the presence of significant amounts of Cu in gold artefacts may reflect an initially high Cu content of the ore^7,64,67,68^ or its deliberate introduction during manufacturing, either to increase the mechanical strength of the object or for coins debasement. In view of these limiting factors, the addition of new isotope data such as Cu and Ag^48,69^ and of detailed ore mineralogy and geochemistry are useful improvements for provenance assessment. Coupling with other isotopic systems, such as copper and silver allows us to refine the tracing and, above all, to highlight different stages of the gold manufacturing. Gold provenance studies using isotopes are very scarce^8,9,11,51,69–71^ nowadays as compared to other metals and most of them do not use archaeological mining referentials, which limits the accurate identification of the ores’ geographical origin^21,72^. Precise archaeological and historical interpretation should rely on the identification of mineralogy and isotope signatures of the ores actually mined by the ancient miners.

The present geochemical work, based on a combined archaeological and geological approach was required to really support the assessment of the economic importance of gold mined by Celtic tribes. It is envisaged that relevant outstanding issues such as the origin of Arvernes gold coinage (a powerful Celtic tribe in Gaul, neighbours of Lemovices tribe and friends of Rome)^73^, or the source of gold used for the Agris helmet (4^th^ c. BC)^74^ would benefit from the present data. The current hypothesis for the origin of the Arvernes gold coinage relies on the recycling of Greek gold coins (notably the Philip II of Macedonia ones)^73^. For the case of the spectacular discovery of the Agris gold helmet in Charente (France), the gold origin for its manufacturing is unknown but some typological and stylistic studies highlighting Balkan-type and/or Etruscan and Greek influences^74^. The age of both the Arvernes coinage and the Agris helmet is contemporary to the period of the gold exploitation of the Gallic tribe of Lemovices. The territorial proximity of the Lemovices mining territory with both the Arvernes living space and the place of discovery of the Agris helmet reinforces the question of the commercial outlet of this important gold production during Celtic times. It may have been local or supra-regional but, more likely, it was integrated into long-distance trade routes in connection with the Phoenicians^26^.

### Conclusions

This study reveals that gold ores mined in Limousin (north-western French Massif Central) during Celtic and modern times respectively are significantly distinct. Their Pb isotope compositions clearly show two different groups of ores, one of Late Proterozoic to Early Paleozoic Pb model age (phase #1), and another one associated to Variscan ages (phase #2). This dichotomy is consistent with field relationships, mineralogy and elemental analyses. Ores with Late Proterozoic to Early Paleozoic model ages may also have been reworked during Variscan tectonics events. According to our understanding of Prehistoric mining activities, Celtic gold production in Limousin belongs to phase #1 and not to phase #2. The negative correlation observed between Cu and Ag isotopic abundances is interpreted as indicating redox phenomena in gossan.

The identification of ore mineralogy and isotope signatures on ores certainly exploited by the ancient miners are needed to refine the isotopic signature of a given ancient mining production district highlighted by archaeometallurgical studies. Future metal provenance studies on Celtic gold artefacts will benefit from this study. Indeed, the use of Cu or Ag isotopes will refine the tracing by the coupling with Pb isotopes and furthermore could highlight humans practices like deliberate additions during metallurgical process or gold coins debasement.

## Methods

### Minerals identification

Grade analysis, reflected light optical microscopy, electron microscopy investigation (SEM) and microprobe analyses (EPMA) were performed on the available ore samples. The SEM observations were carried out on a JSM-6360 electron microscope with a 20 kV voltage, while quantitative chemical analyses were performed using a CAMECA SX50 electron microprobe with an acceleration voltage of 25 kV, a beam current of 20 nA, a surface of the analysed area of 3 × 3 micrometers, and a counting time of 10 s for peaks and 5 s for background.

### Multi elemental analyses

The gold ores were crushed and reduced to fine powder (≤75 µm) and bulk multi-elemental analyses were carried out by ALS Global (www.alsglobal.com). Samples were chemically processed for isotopic analyses in the class 1000 clean room of the Ecole Normale Supérieure de Lyon. All reagents used were double-distilled in PFA stills.

### Multi isotopic analyses

For Cu and Pb isotopes analysis, 50 mg powdered samples were weighted and dissolved in PFA vessels using 2 mL of HNO_3_ and 1 mL of concentrated HF kept at 130 °C for 48 to 72 hours. The solution was then evaporated to dryness and the residue taken back in ~3 mL HCL 6 N, put in ultrasonic bath for 1 hour and set at 130 °C overnight, then evaporated to dryness. This step was repeated. Any residue was taken back in aqua regia, kept at 130 °C for 48 hours, and in case of a persisting residue, the HF-HCl dissolution step was repeated with the dissolution beaker placed in a double-enveloppe metal reactor filled with 500 mL of distilled H_2_O and kept at 160 °C for 12 hours. A last step using 1.5 mL concentrated HNO_3_ and 0.5 mL of Merck® suprapure H_2_O_2_ (30%wt) at 130 °C for 72 hours was eventually used in the rare cases of refractory residues. Final evaporation followed as before.

The sample dissolution for Ag isotopes was carried out in nitric acid and not HCl to avoid AgCl precipitation. 1 mL HF and 3 mL HNO_3_ were added to 75–100 mg of powder. Each beaker was closed and placed on a hot plate at 130 °C for 72 hours. The sample was evaporated to dryness and taken back with ~ 3 mL of concentrated HNO_3_. The solution was sonicated for 1 hour and set at 130 °C overnight. This step was repeated twice. Samples with low Ag concentrations required three separated dissolutions. The samples were taken back in 10 mL HCl 0.1 N and transferred in 10 mL tube for centrifugation.

Isotopic measurements required copper, lead and silver separation by ion exchange. Then, Cu, Pb and Ag were separated from the other elements and purified using the protocols described in Maréchal *et al*.^75^, and Desaulty *et al*.^49^. In order to match our ores matrix, some adjustements were made for Cu, Pb and Ag separation. The samples were eluted on a column filled with 1.6 mL of AG-MP 1 (100–200 mesh, chloride form) anion-exchange resin using 10 mL of 7 M distilled chloride acid and 0.001% H_2_O_2_ to remove the sample matrix, then, by 20 mL 7 M distilled chloride acid and 0.001% H_2_O_2_ to elute Cu, and the procedure was repeated once. The samples were evaporated to dryness and the residue was taken back in ~ 0.2 mL HNO_3_ and 0.1 mL of Merck® suprapure H_2_O_2_ (30%wt) at 110 °C for one hour. Samples were again evaporated to dryness and taken back in 2 mL HNO_3_ 0.5 N and diluted to 300 ng.ml^−1^ in 0.8 mL HNO_3_ 0.05 N for Cu isotopic aquisitions. Pb was separated on an anion-exchange column filled with 0.5 mL AG1-X8 resin (100–200 mesh, chloride form) using 1 M distilled hydrobromic acid to elute the sample matrix and 6 M distilled hydrochloric acid to elute Pb. Samples were evaporated to dryness, diluted to 30 ng.ml^−1^ and taken back in 0.8 mL HNO_3_ 0.05 N for Pb isotopic acquisitions. Ag was separated on an anion-exchange column filled with 1 mL AG1-X8 resin (100–200 mesh, chloride form) using 0.1 M distilled HCl to elute the sample matrix and 9 M distilled hydrochloric acid to elute Ag. After a first elution of Ag, the samples were evaporated to dryness and taken back in 10 mL HCl 0.1 M. This procedure was repeated, then the samples were diluted to 100 ng.ml-1 in HNO_3_ 0.05 M for Ag isotopic acquisitions. Aliquots of purified fractions, for Cu, Pb and Ag chemical separations were monitored by quadrupole-ICP-MS before MC-ICP-MS measurements for yields and for insuring that internal standards were matrix-matched.

Isotopic compositions were measured on the Nu Instruments MC-ICP-MS Nu Plasma HR at Ecole Normale Supérieure de Lyon in the CNRS laboratory. All the details for Cu and Pb isotopic acquisitions are reported in Maréchal *et al*.^75^ and White *et al*.^76^ respectively. The details of the analytical procedure for Ag isotopic acquisition are described in Desaulty *et al*.^49^. Zinc was used to correct for instrumental bias on Cu and Pd for Ag. The values used for reference are those of NIST-SRM 981 Pb reference material reported by Galer and Abouchami (1998)^77^. For Cu, the values are reported relative to the NIST–SRM 976 Cu reference material using the NIST SRM 3114 Cu as a secondary (in-lab) reference. The Ag standard is the NIST-SRM 978a reference material. Copper isotopic data are reported in the conventional δ notation in parts per 1 000 according to the following expressions *i.e* δ^65^Cu = [((^65^Cu/^63^Cu)_sample_/(^65^Cu/^63^Cu)_standard_) − 1] × 1,000 while Ag isotopic data are reported in parts per 10,000 (ε notation), with ε^109^Ag = [((^109^Ag/^107^Ag)_sample_/(^109^Ag/^107^Ag)_standard_) − 1] × 10,000.

The NIST-SRM 981 Pb precision and accuracy of all the reported isotopic ratios are 150 ppm or better. Typical precision for Cu is 50 ppm. Silver isotope measurements were reproduced 5–10 times to achieve a precision of 5–10 ppm.
